# Comparison between subjective perception of trunk deformity (TAPS) and objective assessment of back asymmetry (surface topography)

**DOI:** 10.1186/1748-7161-8-S1-O9

**Published:** 2013-06-03

**Authors:** M Rigo, E D'Agata

**Affiliations:** 1 Institut Elena Salvá, Barcelona, Spain; 2 Fund. Hosp. Univers. Vall D'Hebron-Institut de Recerca. Barcelona, Spain

## Background

The Trunk Appearance Perception Scale (TAPS) is a valid instrument for evaluating the perception that patients have of their trunk deformity [1]. Bagó J et al have showed a significant correlation between TAPS and Cobb angle (r=-.55). Subjective perception has not been compared with an objective method to assess trunk deformity (Formetric 4D).

## Aim

The purpose of this study was to investigate the correlation between subjective perception of trunk deformity and objective evaluation of back asymmetry (Formetric 4D).

## Material and methods

Prospective study including only non operated girls (n=54) diagnosed with IS. Mean age of 14.3+1.4 years (range 12-18). Mean Cobb angle of 31.7º+12.5 (range 10-75). All patients performed the TAPS the same day they were measured with the Formetric system. Radiograph was taken on a different day, and sometimes in a different month, however this was not considered a problem because the TAPS has been already compared with radiographs following a proper methodology. According to our patient evaluation protocol, parents also performed TAPS in a blind way with children. Total sample and subgroups according to curve pattern, single or double as well as according to treatment (RSC brace, other brace type, exercises, no treatment) were analyzed.

## Results

Lateral deviation max and minimums correlated well with the Cobb angle (r=0.76 and r=0.7 respectively) considering both explorations did not coincide in time. Correlation was higher in single than in double curves (lateral deviation max/Cobb angle r=0.83 and r=0.54 respectively), and less also in girls treated with RSC (r=0.45). Correlation between surface rotation max and minimums was lower (r=0.54 and r=0.60 respectively), as expected. TAPS (children) correlated with the Cobb angle not much less (r=-0.47) than previously reported in the study from Bagó et al. Correlation between TAPS and Formetric was even lower than radiological (the higher r=-0.33 with lateral deviation max). TAPS from parents did not correlate with surface topography neither with Cobb angle.

## Conclusion

Correlation between subjective perception of trunk deformity in treated and untreated girls with IS and back asymmetry assessed by surface topography is even lower than that observed with the Cobb angle.
